# A case of autophagia with thalamic hemorrhage

**DOI:** 10.1016/j.amsu.2022.104030

**Published:** 2022-06-28

**Authors:** Nor Osman Sidow, Mohamed Sheikh Hassan

**Affiliations:** Department of Neurology, Somali Mogadişu Türkiye Recep Tayyip Erdoğan Research and Training Hospital, Somalia

**Keywords:** Thalamic, Autophagia, Hemorrhage, Hypertension

## Abstract

Autophagia occurs when one is compelled to inflict pain upon oneself by biting and/or devouring portions of one's body. It is sometimes associated with psychiatric disorders or with acquired nervous system lesions and could be life-threatening (The Journal of Nervous and Mental Disease, February 2012) [2]. It is the first time that this behavior was seen in post-stroke patients, not reported in the medical literature before. A 65-years-old male patient was seen in our emergency department with left side weakness, and agitation that he had self-mutilated his index finger as the same time for one day before admission. He has no previous history of psychiatric illnesses, only he had hypertension. Head CT showed hematoma in the right thalamic and basal ganglia, after orthopedic consultation, the terminal phalange of the index finger in the left hand was amputated, and antipsychotic drugs was started with significant recovery. So thalamic hemorrhage can cause agitation and self mutilating behavior.

Autophagia could be classified under the DSM's Impulse-Control Disorders.Self-mutilation is a severe form of self-injury. Both involve a deliberate and direct injury to one's own body surface without suicidal intent (Claes and Vandereycken, 2007) (Resch et al., 2008) [5].

## 1 Introduction

Autophagia (eating one'sown body) is not classified as a mental disorder or a symptom of a mental disorder in the Diagnostic and Statistical Manual of Mental Disorders (DSM) [1,4]., these behaviors are associated with obsessive-compulsive personality traits. In humans, self-injurious biting behaviors are well described in the setting of mental retardation and psychosis and in persons with Lesch-Nyhan syndrome. Rare cases of human autophagia in persons with intact cognition have been reported, most commonly in the setting of acquired nervous system lesions. After thalamic hemorrhage with self-mutilating behavior, it is the first time being reported.

## 2 Case report

A 65-years-old male patient was seen in our emergency department with left side weakness, altered level of consciousness and agitation for one day. He has no previous of drug, family history including any relevant genetic information, and psychosocial history, only hypertension for two years.

Vital findings were normal *except* for high blood pressure (BP: 204/141). On physical and neurological examination, the *patient's* consciousness was open and agitated. No meningeal irritation sign. Facial asymmetry and left sıde hemiplegia, muscle power was 0,0 in the proximal and distal of upper and lower extremities of the left side. Babiniski sign was positive, and hyper-reflex in the left side. Other system examinations were normal.

**Fig. 1:**
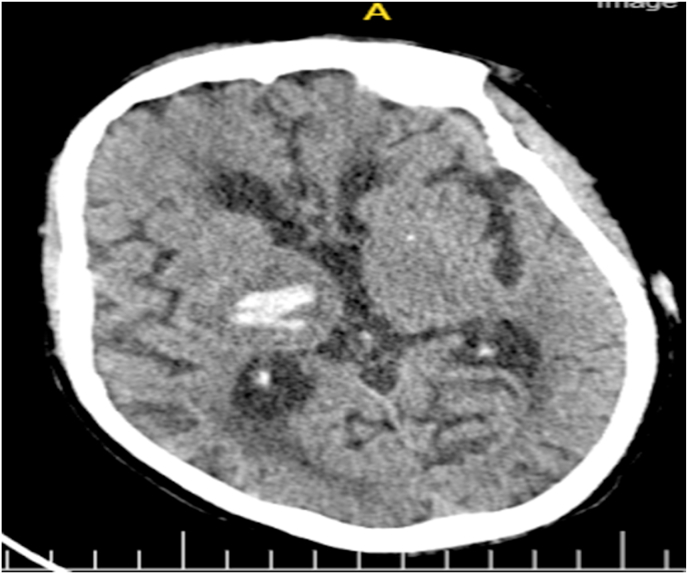
Head CT shows 3 cm diameter hyperdense parenchymal hematoma in the rightperiventriculary and basalganglia. And bifrontal periventricular diffuse hypodensity suggestive of chronic ischemiacchanges

**Fig. 2:**
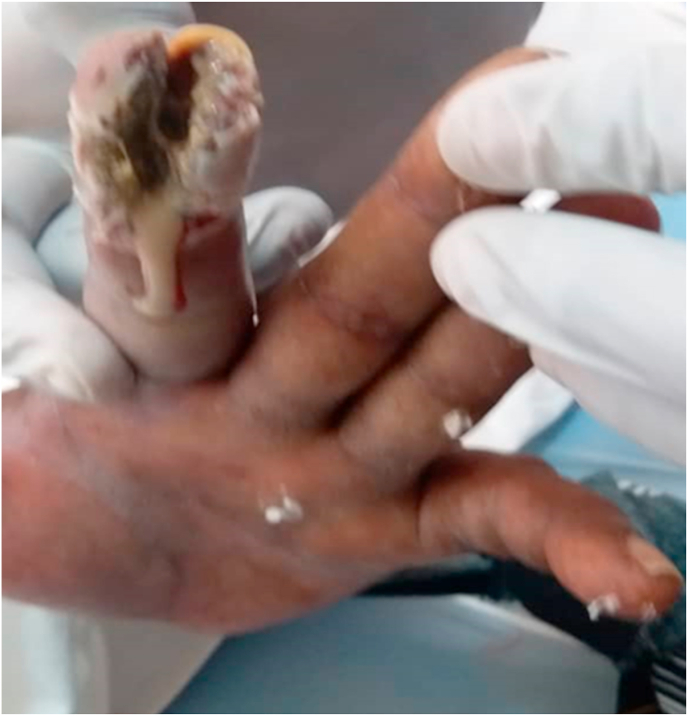
Shows self-mutilatied and chewed the terminal phalange of the index finger in the left hand of the patient for the thalamic hemorrhage.

The patient was diagnosed with hemorrhagic stroke, In the routine biochemistry and CBC performed in our emergency were normal. Pulmonary x-ray also was normal, non-contrast braın CT showed a 3 cm diameter hyperdense parenchymal hematoma in the right periventriculary and basal ganglia level (Fig. 1). The patient was admitted in the neurology department. Treatment of anti brain edema and antihypertensive medications were started.At the same time of hospital admission he had mutilated his finger with agitation until he chewed the terminal phalange of his index fingerin the left hand (Fig. 2). We started haloperidol 5 oral drops during agitation and added with olanzapine (10 mg/day).

The self-mutilating behavior slightly decreased, at the same time we did orthopedic consultation, and they recommended amputation for the finger. After the patient's condition became stable orthopedic did amputation. We repeated the brain CT, the hematoma decreased which revealed a 2,5 cm diameter hyperdense parenchymal hematoma in the right periventriculary and basal ganglia level. So we decided to discharge the patient and come dressing daily with orthopedic department, and control for two weeks later for neurology department.

## 3 Discussion

Self-mutilation is a severe form of self-injury. Both involve a deliberate and direct injury to one's own body surface without suicidal intent (Claes and Vandereycken, 2007) [3].So, this patient didn't have any mental illness history before, and developed this behavior -mutilated his finger with agitation until he chewed the terminal phalange of his index fingerin the left hand-after one day of thalamic hemorrhage, after a couple days with haloperidol 5 oral drops during agitation and added with olanzapine (10 mg/day),he became stable. It is not reported for post-stoke patients with self mutilating behavior before in the medical literature. We report here a case of autophagia with thalamic hemorrhage. This case has been reported in line with the SCARE 2020 criteria [6].

In the literature, it is first time to report like this case for a patient with thalamic hemorrhage who injured himself without known any psychosocial history or mental illness.

The limitation of the study; there was no previous same case reports in the literature to compare our case report.

## 4 Conclusion

It is the first time being seen in medical literature for a thalamic hemorrhagic patient with self-mutilating behavior. We amputated the patient's finger and started antipsychotic drugs which proved to be beneficial. We speculate that this behavior is due to the thalamic hemorrhage.

## Ethical approval

No ethical Approval is needed for this case series.

## Sources of funding

There is no any funding for this case series.

## Author contribution

Corresponding writer, Nor Osman Sidow, writing and publication of the case series, Co-author, Mohamed Sheikh Hassan, design and interpretation of the case series, N/A.

## Declaration of competing interest

All authors have no any personal or financial interest for publication of this case series.

## Registration of research studies

No Registry was done.

## Guarantor

Nor Osman Sidow.

## Consent

Written informed consent was obtained from the patient for publication of this case series and accompanying images and is available for review by the Editor-in-Chief of this journal on request.

## Provenance and peer review

Not commissioned, externally peer-reviewed.

## References

[1] American Psychiatric Association (2000).

[3] Sheehy E.C., Longhurst P., Pool D., Dandekar M. (1999). Self-inflictedinjury in a case of Hallervorden-Spatz disease. Int. J. Paediatr. Dent..

[4] Pattij T., Vanderschuren L.J. (2008). The neuropharmacology of impulsive behaviour. Trends Pharmacol. Sci..

[6] Agha R.A., Franchi T., Sohrabi C., Mathew G., for the SCARE Group (2020). The SCARE 2020 guideline: updating consensus surgical CAse REport (SCARE) guidelines. Int. J. Surg..

